# Rotavirus-Specific Maternal Serum Antibodies and Vaccine Responses to RV3-BB Rotavirus Vaccine Administered in a Neonatal or Infant Schedule in Malawi

**DOI:** 10.3390/v16091488

**Published:** 2024-09-19

**Authors:** Benjamin Morgan, Eleanor A. Lyons, Amanda Handley, Nada Bogdanovic-Sakran, Daniel Pavlic, Desiree Witte, Jonathan Mandolo, Ann Turner, Khuzwayo C. Jere, Frances Justice, Darren Suryawijaya Ong, Rhian Bonnici, Karen Boniface, Celeste M. Donato, Ashley Mpakiza, Anell Meyer, Naor Bar-Zeev, Miren Iturriza-Gomara, Nigel A. Cunliffe, Margaret Danchin, Julie E. Bines

**Affiliations:** 1 Enteric Diseases, Murdoch Children’s Research Institute, Parkville, VIC 3052, Australiaamanda.handley@mcri.edu.au (A.H.); nada.bogdanovic@mcri.edu.au (N.B.-S.); daniel.pavlic@mcri.edu.au (D.P.); fran.justice@mcri.edu.au (F.J.); darren.ong@mcri.edu.au (D.S.O.); rhian.bonnici@mcri.edu.au (R.B.); celeste.donato@mcri.edu.au (C.M.D.); margie.danchin@mcri.edu.au (M.D.); 2 Medicines Development for Global Health, Melbourne, VIC 3001, Australia; 3 Malawi Liverpool Welcome Trust Programme, Blantyre P.O. Box 30096, Chichi, Malawi; dwitte@mlw.mw (D.W.); jmandolo@mlw.mw (J.M.); khuzwayo.jere@liverpool.ac.uk (K.C.J.);; 4 Institute of Infection, Veterinary and Ecological Sciences, University of Liverpool, Liverpool L69 7ZX, UK; amturner@liverpool.ac.uk (A.T.); m.iturriza-gomara@liverpool.ac.uk (M.I.-G.); n.a.cunliffe@liverpool.ac.uk (N.A.C.); 5 Department of Clinical Sciences, Liverpool School of Tropical Medicine, Liverpool L3 5QA, UK; 6 Department of Gastroenterology and Clinical Nutrition, Royal Children’s Hospital, Parkville, VIC 3052, Australia; 7 GSK Vaccines for Global Health Institute, 53100 Sienna, Italy; 8 Department of General Medicine, Royal Children’s Hospital, Parkville, VIC 3052, Australia; 9 Department of Paediatrics, The University of Melbourne, Parkville, VIC 3052, Australia

**Keywords:** rotavirus vaccine, maternal antibodies, RV3-BB vaccine

## Abstract

High titres of rotavirus-specific maternal antibodies may contribute to lower rotavirus vaccine efficacy in low- and middle-income countries (LMICs). RV3-BB vaccine (G3P[6]) is based on a neonatal rotavirus strain that replicates well in the newborn gut in the presence of breast milk. This study investigated the association between maternal serum antibodies and vaccine response in infants administered the RV3-BB vaccine. Serum was collected antenatally from mothers of 561 infants enrolled in the RV3-BB Phase II study conducted in Blantyre, Malawi, and analysed for rotavirus-specific serum IgA and IgG antibodies using enzyme-linked immunosorbent assay. Infant vaccine take was defined as cumulative IgA seroconversion (≥3 fold increase) and/or stool vaccine shedding. Maternal IgA or IgG antibody titres did not have a negative impact on vaccine-like stool shedding at any timepoint. Maternal IgG (but not IgA) titres were associated with reduced take post dose 1 (*p* < 0.005) and 3 (*p* < 0.05) in the neonatal vaccine schedule group but not at study completion (week 18). In LMICs where high maternal antibodies are associated with low rotavirus vaccine efficacy, RV3-BB in a neonatal or infant vaccine schedule has the potential to provide protection against severe rotavirus disease.

## 1. Introduction

Rotavirus vaccines are recommended for all children and have been introduced in 126 countries worldwide, providing a cost-effective pathway to reduce hospitalization and death among young children [1,2]. However, despite this significant achievement, rotavirus gastroenteritis remains the most common cause and still accounts for a quarter of global diarrheal deaths in children less than 5 years of age [3]. Barriers to the success of rotavirus vaccines include challenges to vaccine access (such as cost, supply, health prioritization, and service delivery), timely administration, and vaccine safety concerns [1,4]. A significant disparity in vaccine efficacy and effectiveness has been observed for the licensed vaccines Rotarix^®^ (GlaxoSmithKline, Rixensart, Belgium) and RotaTeq^®^ (Merck, Whitehouse Station, NJ, USA) when rotavirus vaccines have been implemented in low- and middle-income countries (LMICs) compared to high-income countries (HICs) [5]. The median vaccine effectiveness of the Rotarix^®^ vaccine is estimated at 57% when implemented in LMICs compared to 84% in HICs. A similar pattern has been reported with the RotaTeq^®^ vaccine at 45% and 90% for LMICs and HICs, respectively [6]. It has been postulated that co-administration with oral polio vaccine, interference in replication by other enteric pathogens, immaturity of the immune system, and malnutrition could contribute to this disparity [5,6,7]. 

**Figure 1:**
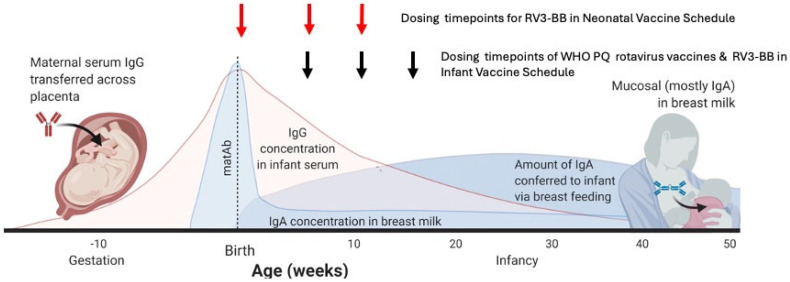
Timing of administration of rotavirus vaccines in association with age-related changes in titres of maternal serum and breastmilk antibodies and infant serum IgG and IgA titres. Neonatal vaccine schedule (first dose at birth) (red arrows) with RV3-BB vaccine; infant vaccine schedule (first dose at 6–8 weeks of age) (black arrows): WHO-prequalified rotavirus vaccines (Rotarix^®^, RotaTeq^®^, Rotavac^®^ (Bharat Biotech, Hyderabad, India) and Rotasiil^®^ (Serum Institute of India, Pune, India) and RV3-BB vaccine administered in the infant schedule). Adapted from: Otero CE, Langel SN, Blasi M, Permar SR. PLoS Pathogens 2020 16(11):e1009010 [8].

Maternal serum (IgG) and breast milk (IgA) antibodies are critical to provide passive immunity while infants establish their own immune defences and have been proposed as a major factor contributing to lower rotavirus vaccine efficacy in LMICs [5,7,8,9,10]. Serum IgG antibodies are transferred across the placenta and provide systemic passive immunity for a newborn during their first few months of life, with high titres commonly observed at birth followed by a decline over time, with a half-life of 3 to 4 weeks (Figure 1) [8]. The rate of this decline is influenced by the initial antibody titre, maternal health and nutrition, and exposure to wild-type infection [8,11]. High titres of maternal serum rotavirus-specific IgG antibodies have consistently been associated with reduced immunogenicity after administration of rotavirus vaccines and have been proposed to be associated with reduced vaccine efficacy in LMICs [8,9,12,13]. Serum IgA antibodies are detected in maternal serum but not transmitted transplacentally. Infants rely on maternal breast milk IgA to provide local immunity to the gut mucosa and support protection from enteric pathogenic bacteria and viruses [8]. Reduced immunogenicity in response to a rotavirus vaccine was reported in infants of mothers with high rotavirus-specific IgA titres in breast milk in Vietnam and Zambia but not in Nicaragua [12,13,14]. However, clinical trials of withholding breastfeeding (30 min to 60 min) prior to administration of the Rotarix^®^ vaccine in South Africa, Pakistan, and India failed to show a difference in seroconversion in those infants who had breastfeeding withheld compared to those who did not [15,16,17]. 

Administration of a rotavirus vaccine at birth or soon after birth has the potential to address some of the current challenges to vaccine implementation and to improve the performance and safety of a rotavirus vaccine [18]. However, administration of an oral rotavirus vaccine from birth maximises exposure to high levels of maternal serum IgG antibodies and breast milk IgA [8] (Figure 1). The RV3-BB rotavirus vaccine is based on an asymptomatic neonatal rotavirus strain (RV3: G3P[6]) that replicates well in the newborn gut despite the presence of breast milk [19,20]. Clinical trials have shown that RV3-BB is well tolerated in a neonatal vaccine schedule, is immunogenic, and was associated with a vaccine efficacy of 75% against severe rotavirus disease at 18 months in Indonesia [21,22]. In clinical trials in New Zealand and Indonesia, there was no association between maternal rotavirus-specific IgG antibody titre, colostrum or breast milk IgA antibodies, and vaccine take, serum IgA response, or stool vaccine virus shedding after three doses of RV3-BB vaccine [23,24]. 

Infants living in Malawi are often exposed to rotavirus infection early in life [25]. High titres of maternal rotavirus-specific antibodies have been reported in mothers in Malawi and were negatively correlated with response to Rotarix^®^ vaccine, in particular vaccine virus shedding [7]. The primary aim of this study was to determine whether maternal serum rotavirus-specific IgA and IgG antibody titres in mothers in Malawi are associated with vaccine take (serum IgA response and/or vaccine virus shedding) in their infants following administration of the RV3-BB rotavirus vaccine in a neonatal vaccine schedule (with the first dose within 5 days of birth) or infant vaccine schedule (with the first dose administered at 6 weeks of age). 

## 2. Materials and Methods

### 2.1. Study Design and Participants

**Figure 2:**
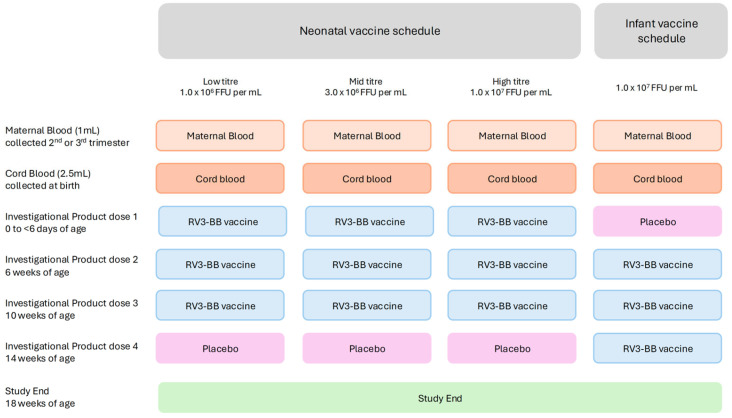
Study design.

The Maternal Antibody study was an exploratory analysis nested within the Phase II RV3-BB trial, a randomized, double-blind, placebo-controlled four-arm parallel-group, dose-ranging study of oral human neonatal rotavirus vaccine (RV3-BB) administered at a titre of 1.0 × 10^6^ FFU per mL (low-titre group), 3.0 × 10^6^ FFU per mL (mid-titre group), or 1.0 × 10^7^ FFU per mL (high-titre group) as a three-dose neonatal schedule, or administered at a titre of 1.0 × 10^7^ FFU per mL as a three-dose infant schedule (Figure 2) [26]. The study was conducted between September 2018 and January 2020, and involved 711 infants recruited from three primary healthcare centres in Blantyre, Malawi [26]. All mothers of the 711 eligible participants recruited to the Phase II study were invited to participate in the Maternal Antibody study. A two-stage consent process was followed. Pregnant women provided consent for the collection of a pre-birth maternal blood and stool sample and an after-birth infant cord blood and stool sample. Written informed study consent was obtained after birth from parents or guardians. Study exclusions were the same as for the main study [26]. Participants from the Phase II study per protocol population who received three doses of vaccine according to the protocol and with maternal blood samples available for analysis were included in the Maternal Antibody study.

The study was conducted in accordance with the International Council for Harmonization of Good Clinical Practice Guidelines. The protocols were approved by the Ethics Committees of the Royal Children’s Hospital Melbourne and the University of Liverpool, the National Health Science Research Committee, and the Pharmacy and Medicines and Poisons Board of Malawi. This trial is registered at ClinicalTrials.gov (NCT03483116).

### 2.2. Sample Collection and Processing

Maternal venous blood (5–10 mL) was collected in the second or third trimester of pregnancy. Serum was isolated from whole blood and stored at −70 °C until analysed. Blood was collected from the cord (baseline for neonatal schedule comparison) immediately before IP dose 2 (baseline for infant schedule comparison), 28 days after IP dose 3, and 28 days after IP dose 4. A pre-dose infant blood sample was also collected at IP doses 2, 3, and 4 and at 18 weeks of age [26]. The serum was frozen at −70 °C and shipped to the Murdoch Children’s Research Institute laboratory for analysis. Rotavirus-specific IgA and IgG antibody titres were measured by enzyme-linked immunosorbent assay (ELISA) using rabbit anti-RV3 polyclonal antisera as the coating antibody and RV3-BB virus or Vero cell lysate as the capture antigen [27]. The antigen–antibody complexes were detected with biotinylated anti-human IgA and streptavidin–horseradish peroxidase [27]. Concentrations of rotavirus-specific IgA were measured using a standard curve generated from known positive serum samples arbitrarily assigned a titre of 250,000 units per millilitre (U/mL). The lower limit of detection was 20 U/mL.

Rotavirus shedding was assessed in stool samples collected at baseline, before administration of the first dose of IP, and between days 3 to 7 after administration of each dose of IP. Samples were assessed using a rotavirus VP6-specific reverse transcriptase polymerase chain reaction (PCR). PCR products were analysed by electrophoresis with the Invitrogen one-step RT-PCR key (Invitrogen, Carlsbad, CA, USA) and Rot3 and Rot5 oligonucleotide primers [28]. Sequence analysis was used to confirm the presence of the RV3-BB vaccine (Sequencher Software program version 4.1, Gene Codes Corp Inc., Ann Arbor, MI, USA) and identity determined by GenBank database [28]. 

### 2.3. Definition of Vaccine Response

IgA seroconversion was defined as serum anti-rotavirus IgA antibody titre equal to or greater than three times the titre of the baseline titre and was assessed after each dose of IP. Stool shedding was defined as the presence of RV3-BB vaccine-like virus detected in the stool collected between 3 to 7 days after a dose of IP. Vaccine take was defined as a serum immune response (≥3-fold increase in titre from baseline) of anti-rotavirus IgA 28 days following IP administration, and/or the presence of vaccine-like virus in stool 3 to 7 days following IP administration [26]. Cumulative vaccine serum response, stool shedding, and/or vaccine take was defined as a positive result following one, two, or three IP doses for the neonatal vaccine group, and following two, three, or four IP doses for the infant vaccine schedule group.

### 2.4. Statistical Analysis

Demographic characteristics of participants are presented using means and standard deviations (SDs) for continuous variables and proportions for categorical variables. Mean maternal serum IgA and IgG titres are presented as log (natural) transformed data. Infant serum IgA response, stool excretion, and cumulative vaccine take are presented as numbers and proportions for each schedule group and the combined neonatal vaccine group (low-titre, mid-titre, and high-titre neonatal groups combined). Separate linear regression models were used to explore the relationship between maternal serum IgA and IgG titres against infant anti-rotavirus serum IgA antibody titre and seroconversion after dose 1 and dose 3. Statistical analyses were performed with Prism (GraphPad Software, Inc., La Jolla, CA, USA, version 10.0.1) by use of the unpaired Mann–Whitney test due to data not fitting the normal distribution. A *p* value of less than 0.05 was considered to be significant. 

## 3. Results

### 3.1. Study Population

**Figure 3:**
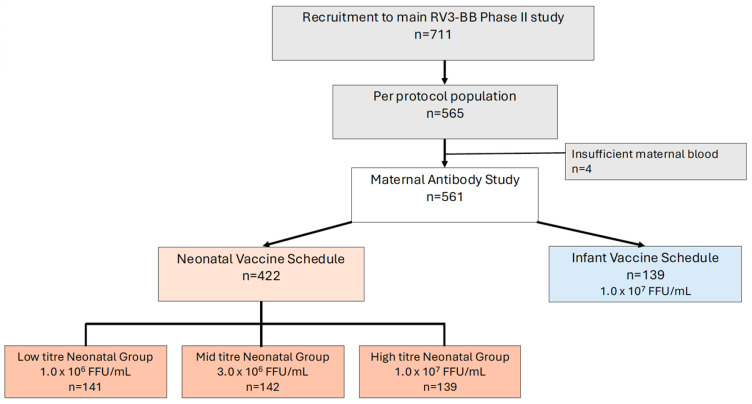
Infant participant flow for the Maternal Antibody study.

A total of 711 infants were recruited to the Phase II study. Of these, 565 participants received three doses of the RV3-BB vaccine according to the protocol, therefore fulfilling criteria for inclusion in the per-protocol analysis population [26]. All 565 infants were eligible to be included in the Maternal Antibody study; however, four mother–infant pairs were excluded, as insufficient or no blood was available from the mother for analysis, resulting in an analysis study population of 561 infants (Figure 3). There were no significant differences in characteristics between infants recruited to the Maternal Antibody study compared to the Phase II study, or between treatment allocation groups (Table 1). 

### 3.2. Maternal Antibodies and RV3-BB Vaccine Response

#### 3.2.1. Maternal Antibodies and RV3-BB Vaccine Stool Shedding

**Figure 4:**
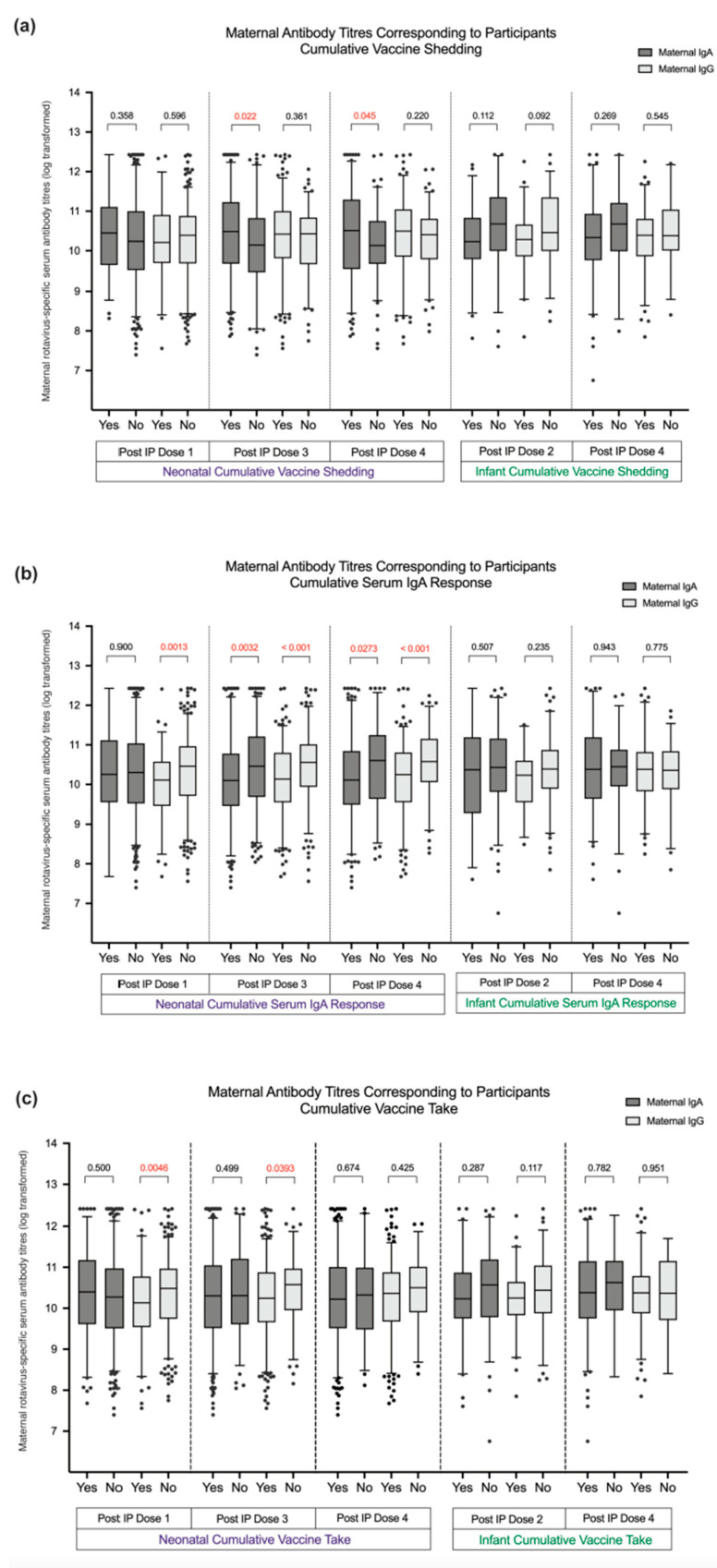
(**a**–**c**) Mean maternal rotavirus-specific serum IgA and IgG antibody titres (log transformed) in association with vaccine response in participants administered RV3-BB vaccine in the combined neonatal vaccine schedule group and the infant vaccine schedule group. The “y” axis denotes the serum maternal serum IgA and IgG antibodies titres (log). The “x” axis denotes the study groups according to neonatal or infant vaccine schedule group, per Investigation product (IP) dose, and according to vaccine response with the positive vaccine response variable (“Yes”) or negative vaccine response variable (“No”). Data are presented in a box-and-whisker plot, with the box extending from the 25th to the 75th percentile and the line in the middle plotted at the median. The whiskers represent the 10–90th percentiles, with all datapoints outside the 10–90th percentiles shown. Statistics and plotting were performed in Prism 9 for MacOs. Non-normally distributed data were analysed using the Mann–Whitney test. 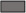 Maternal rotavirus-specific serum IgA antibody titres (log). 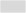 Maternal rotavirus-specific serum IgG antibody titres (log).

Maternal rotavirus-specific IgA and IgG antibody titres were not negatively associated with RV3-BB vaccine virus shedding in the stool at any timepoint in the neonatal and infant vaccine schedule. A positive association between high maternal IgA titres and stool shedding observed after the third dose of RV3-BB vaccine (IP dose 3) and at study completion at week 18 (IP dose 4) in the neonatal vaccine schedule (Figure 4a). 

#### 3.2.2. Maternal Antibodies and RV3-BB Vaccine Anti-Rotavirus IgA Seroconversion 

**Figure 5:**
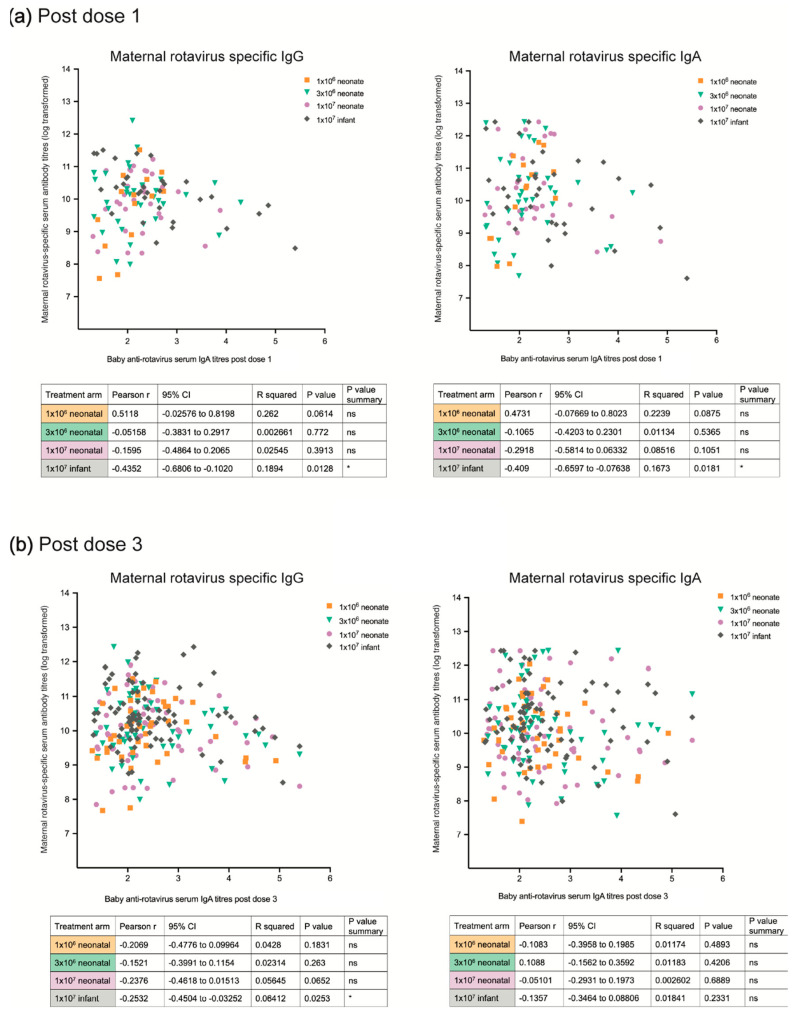
Maternal rotavirus-specific serum IgA and IgG antibody titres are plotted against the anti-rotavirus serum IgA antibody titres of their infant after (**a**) the first dose (post vaccine dose 1) and (**b**) the full three-dose course (post vaccine dose 3) in each of the four treatment allocation groups. Separate linear regression models were used to explore the relationship between maternal rotavirus-specific serum IgA and IgG antibody titres (log transformed) against infant anti-rotavirus serum IgA antibody titre after vaccine dose 1 and dose 3. Statistical analyses were performed with Prism (GraphPad Software, Inc., version 10.0.1) by use of the unpaired Mann–Whitney test due to data not fitting the normal distribution. A *p* value of less than 0.05 was considered to be significant. 95% CI = 95% confidence interval. * = significant *p* < 0.05. ns = not significant *p* > 0.05. 1.0 × 10^6^ neonate = low-titre neonatal vaccine schedule group. 3.0 × 10^6^ neonate = mid-titre neonatal vaccine schedule group. 1.0 × 10^7^ neonate = high-titre neonatal vaccine schedule group. 1.0 × 10^7^ infant = infant vaccine schedule group.

Maternal serum IgA antibody titres were inversely associated with anti-rotavirus IgA seroconversion in infants after three doses of RV3-BB vaccine (IP dose 3 and IP dose 4 in the combined neonatal group (Figure 4b) and in the high-titre neonatal vaccine schedule group (*p* = 0.03) (Supplementary Table S1). High maternal IgG antibody titres were associated with a lower proportion of infants who had anti-rotavirus serum IgA seroconversion after one dose (IP dose 1) and three doses of RV3BB vaccine (IP dose 3 and IP dose 4) administered in the neonatal vaccine schedule group (*p* = 0.0013, *p* < 0.001, and *p* < 0.001, respectively) (Figure 4b). This was also observed when analysed in the three vaccine titre groups (Supplementary Table S1). No significant association was observed between maternal rotavirus-specific serum IgA or IgG antibody titres (log transformed) and anti-rotavirus serum IgA antibody titres in their infant after dose 1 or dose 3 in the neonatal vaccine schedule (Figure 5). In contrast, there was no significant association observed between maternal serum IgA or IgG antibody titres and IgA seroconversion in infants administered RV3-BB in the infant vaccine schedule (Figure 4b). However, a significant association was observed between maternal serum IgA and IgG antibody titres and the anti-rotavirus serum IgA titres of their infant after vaccine dose one (IP dose 2), and with maternal serum IgG antibody titres and anti-rotavirus serum IgA antibody titres in their infant after the third vaccine dose (IP dose 4) (Figure 5). 

#### 3.2.3. Maternal Antibodies and RV3-BB Vaccine Take

Maternal rotavirus-specific IgA antibody titres were not significantly different in participants with a positive or negative vaccine take after dose 1 or 3 of vaccine in the combined neonatal schedule group (IP dose 1 and IP dose 3) or at study completion at 18 weeks of age (IP dose 4) (Figure 4c). In the neonatal vaccine schedule group, there were no significant differences observed when analysed according to vaccine titre groups (Supplementary Table S1) or in the infant schedule group (*p* > 0.05 for all comparisons) (Supplementary Table S1). An association between high maternal IgG antibody titres and vaccine take in the infant were observed after dose 1 and dose 3 in the combined neonatal vaccine schedule group (*p* = 0.005 and *p* = 0.04, respectively), but this association was not sustained at study completion at 18 weeks of age (IP dose 4) (*p* > 0.05) (Figure 3). No association between maternal IgG antibody titre and vaccine take in the infant was observed when analysed according to vaccine titre group (*p* > 0.05 for all comparisons) or in the infant schedule group (*p* > 0.05) (Supplementary Table S1). 

## 4. Discussion

In this study, maternal rotavirus-specific serum IgA antibody titres did not reduce the take of the RV3-BB rotavirus vaccine when administered in a neonatal vaccine schedule (with the first dose at birth) or an infant vaccine schedule (first dose at 6 weeks of age) in infants in Malawi. High maternal IgG antibodies reduced take after one and three doses of vaccine in the neonatal schedule, although this was not observed at study end (week 18), suggesting that any inhibition was not sustained. Stool shedding of vaccine virus was not impacted by high maternal rotavirus-specific IgA or IgG antibody titres. The results of this study reflect findings of previous RV3-BB clinical trials conducted in New Zealand and Indonesia and suggests that this finding is not specific to a region or a population [23,24].

High maternal rotavirus-specific serum and breastmilk antibody levels have been reported in a number of studies from LMICs, including from India, Nicaragua, Mexico, Indonesia, and South Africa, compared to mothers from the USA [7,8,9,10,12,13,14,15,29,30]. The inverse relationship between serum maternal IgG and/or IgA antibody titres and infant immune responses has previously been reported following administration of rotavirus vaccines [8,9,13]. High maternal pre-vaccination serum IgG antibody titres were negatively associated with infant seroconversion (*p* = 0.031) and infant serum IgA antibody titres following administration of the first dose of the Rotarix^®^ vaccine in South African infants, although this inhibition was overcome by the second dose of the vaccine [30]. Similar observations between rotavirus-specific maternal IgG antibody titres and infant seroconversion were reported following the first dose of the RotaTeq^®^ vaccine in infants from Nicaragua (*p* = 0.02) [12] and after three doses of the Rotavac^®^ vaccine in infants in India [31]. Although we observed a negative association between the titres of maternal antibody and anti-rotavirus IgA antibody titres in the infant vaccine schedule, this did not translate to a lack of anti-rotavirus IgA seroconversion in these infants. It has been proposed that the negative relationship between high maternal antibodies in mothers from LMICs and infant serum IgA response after the first vaccine dose but not consistently observed after a complete two- or three- dose vaccine schedule likely relates to the normal exponential decline in transplacental IgG antibodies [24,25,32]. Delaying the first dose of a rotavirus vaccine schedule in an effort to avoid the inhibitory effect of maternal antibodies has been suggested, but this would leave young infants unprotected, particularly in high rotavirus disease burden regions in LMICs where severe rotavirus disease still occurs, despite the presence of high titres of maternal antibodies. Delaying the administration of rotavirus vaccines also may have other implications, including an increased risk of vaccine-associated intussusception [33]. 

Maternal IgG antibodies have been associated with an inhibition of seroconversion with all vaccine types for a range of viral and bacterial pathogens—not only live-attenuated vaccines but also inactivated, subunit, and protein vaccines [11]. Inhibition of vaccine responses by maternal antibodies has been reported for a number of childhood vaccines, including measles and polio vaccine [11]. It has been proposed that inhibition of vaccine antigen-specific B-cell activation by maternal IgG occurs via the development of a vaccine–antibody complex involving cross-linking the B-cell receptor and the inhibitory/regulatory Fcγ-receptor IIB and/or epitope masking, neutralization of vaccine virus, and removal of vaccine antigen by phagocytosis [11]. But if, or how, these mechanisms may differ in children in LMICs compared to children in HICs is not known. 

The lack of an accurate serologic correlate of protection presents a challenge in comparing results between rotavirus vaccine studies [34,35]. Anti-rotavirus serum IgA antibody levels after acute infection are currently considered the best serological marker of protection; however, they are an indirect marker, as intestinal IgA is considered as the key mechanism for clearance of infection [36,37]. Anti-rotavirus serum IgA response is commonly used as a serological marker of immunogenicity in clinical vaccine trials, although the laboratory method for analysis and definitions of vaccine response vary across vaccine studies [38]. Higher titres of infant serum IgA correlate with a lower risk of rotavirus infection, although this is not always a consistent measure of protection against rotavirus disease across studies [34,35,38]. Rotavirus serum-neutralising antibodies have also been measured as a serum marker of vaccine response [34,35]. In New Zealand infants, serum-neutralising antibody responses were not impacted by colostrum or breastmilk IgA titres after three doses of RV3-BB vaccine [23]. Although not validated as a correlate of protection, vaccine virus shedding detected in the stool after immunisation with a rotavirus vaccine reflects gut replication of the vaccine virus and has been proposed as a marker of the mucosal immune response [7,21,22,26,39]. In a comparative study of rotavirus vaccine responses in Malawi and India, the negative correlation between maternal rotavirus-specific serum and breastmilk IgA antibodies and rotavirus vaccine response was suggested to be driven by a reduction in vaccine virus replication and shedding [7]. Interestingly, in the United Kingdom cohort in the same study, vaccine virus shedding was not inhibited in the presence of similar titres of maternal serum IgA antibodies [7]. In contrast, our study did not identify a negative impact on RV3-BB vaccine virus shedding by high maternal antibody titres. On the contrary, increased shedding was observed in the presence of high maternal IgA antibody titres in the neonatal vaccine schedule group. The RV3-BB vaccine is a human, neonatal rotavirus vaccine based on the naturally attenuated, asymptomatic human neonatal strain (RV3: G3P[6]). Neonatal P[6] strains are phenotypically different from pathogenic wildtype rotavirus strains [40]. The VP4 outer capsid proteins of neonatal P[6] rotavirus strains differ at a number of amino acid positions compared to pathogenic strains isolated from older infants and children. The wildtype RV3 strain has six amino acid changes located on the basal surface of the VP8* core, which sits outside the putative neutralization domain [40]. It has been proposed that these changes may allow neonatal strains to bind more efficiently to carbohydrate molecules or proteins found on enterocytes in the newborn gut and potentially evade neutralisation by maternal IgG antibodies [40]. Importantly, in Indonesia, the RV3-BB vaccine provided robust protection against severe rotavirus disease in the first 18 months of life (75%) when administered in the neonatal vaccine schedule, despite a negative association observed between cord blood IgG antibody titres and serum IgA seroconversion and vaccine take after the first dose [21,24].

A limitation of this study reflects the lack of a perfect serological surrogate for protection against rotavirus infection or for the assessment of the response to a rotavirus vaccine. To address this limitation, we have presented results of “vaccine take,” which takes into account anti-rotavirus IgA seroconversion and vaccine virus shedding in the stool [21,22,26]. To understand the characteristics of the influence of maternal antibodies on infant vaccine response, it would be ideal to measure maternal antibodies at a number of time points antenatally, during labour and cord blood, and also to assess a broader panel of immune factors. Unfortunately, this was not feasible within the confines of this study. As serum maternal IgA antibodies are not transferred via the placenta, breastmilk IgA antibodies would have also provided further information but were not measured in the current study. However, we did not find an association between IgA antibodies in colostrum or breastmilk and vaccine take after three doses of RV3-BB vaccine when administered in either the neonatal or infant vaccine schedules in New Zealand or Indonesia [23,24].

In summary, maternal rotavirus-specific IgG (but not IgA) antibody titres were associated with reduced vaccine take at after dose 1 and dose 3 in the neonatal vaccine schedule group but not at study completion (week 18). Maternal IgG and IgA antibody titres were inversely associated with IgA seroconversion in the neonatal vaccine schedule group but had no impact on vaccine shedding at any timepoint in either schedule. These results are consistent with observations from RV3-BB vaccine studies conducted in Indonesia and New Zealand, where there was no association between IgA in colostrum and breastmilk and vaccine take after three doses of RV3-BB vaccine when administered in the neonatal or infant vaccine schedules. Using a neonatal vaccine schedule RV3-BB vaccine has the potential to address the disparity in rotavirus vaccine performance that currently persists in LMICs.

## Figures and Tables

**Table 1:** Infant participant characteristics.

	Neonatal Vaccine Schedule (*n* = 422)			Infant Vaccine Schedule (*n* = 139)
	Low Titre	Mid Titre	High Titre	
	(*n* = 141)	(*n*=142)	(*n* = 139)	(*n* = 139)
Gestational age (weeks): mean (min, max)	37.5 (34, 41)	37.3 (36, 40)	37.6 (35, 43)	37.6 (36, 45)
Birth weight (grams): mean (SD)	3121.9 (359.59)	3117.9 (384.39)	3089.4 (344.49)	3150.8 (362.65)
Sex (male): number (%)	70 (49.6)	67 (47.2)	81 (58.3)	74 (53.2)
Ethnicity (Black African): number (%)	141 (100)	142 (100)	139 (100)	139 (100)
Exclusive breastfeeding duration: number (%)				
Day 1–6	141 (100)	142 (100)	139 (100)	139 (99.3)
Week 1–6	141 (100)	142 (100)	139 (98.6)	139 (99.3)
Week 10	141 (100)	142 (100)	139 (98.6)	139 (100)
Week 14	139 (98.6)	140 (97.9)	139 (98.6)	139 (100)

## Data Availability

Data are available on request.

## Supplementary Materials

The following supporting information can be downloaded at: https://www.mdpi.com/article/10.3390/v16091488/s1, Table S1: Comparisons between maternal rotavirus-specific IgA and IgG antibodies titres (log transformed) and vaccine responses in their infants who had been administered RV3-BB in the neonatal vaccine schedule group presented according to vaccine titre groups.
