# Supplementary material for: Rotavirus-Specific Maternal Serum Antibodies and Vaccine Responses to RV3-BB Rotavirus Vaccine Administered in a Neonatal or Infant Schedule in Malawi
Source: Viruses. 2024 Sep 19;16(9):1488. doi: 10.3390/v16091488 (PMC11437397; doi:10.3390/v16091488)
Supplement: Supplementary file 1 [file viruses-16-01488-s001.zip › viruses-3185652-Supplementary Table S1.pdf]

Supplementary Table S1

Comparisons between maternal rotavirus-specific IgA and IgG antibodies titres (log transformed) and vaccine responses in their infants who had been administered RV3-BB in the neonatal vaccine schedule group presented according to vaccine titre groups.

Significant comparisons *p<0.05. **p<0.005

	NEONATAL VACCINE SCHEDULE LOW TITRE GROUP		NEONATAL VACCINE SCHEDULE MID TITRE GROUP		NEONATAL VACCINE SCHEDULE HIGH TITRE GROUP	
**Maternal serum rotavirus specific IgA antibody:**						
Mother-baby pairs (n)	141		142		139	
Maternal serum IgA Geometric mean titre log transformed (95% CI)	10.28 (10.10, 10.46)		10.25 (10.06, 10.44)		10.34 (10.17, 10.51)	
**Maternal Serum rotavirus specific IgG antibody:**						
Mother-baby pairs (n)	140		141		139	
Maternal serum IgG Geometric mean titre log transformed (95% CI)	10.29 (10.11, 10.47)		10.16 (9.94, 10.38)		10.12 (9.88, 10.36)	
	**VACCINE RESPONSES IN INFANTs ADMINISTERED RV3-BB ACCORDING TO VACCINE TITRE GROUP**					
	**VACCINE TAKE**		**VACCINE TAKE**		**VACCINE TAKE**	
	**Yes**	**No**	**Yes**	**No**	**Yes**	**No**
**Neonatal vaccine schedule post-dose 1**						
Maternal serum rotavirus specific IgA:						
Mother-baby pairs (n)	23	118	45	97	50	89
Maternal serum IgA Geometric mean titre log transformed (95% CI)	10.23 (1.12)	10.29 (1.09)	10.23 (1.18)	10.26 (1.14)	10.51 (1.17)	10.24 (0.94)
Maternal IgG:						
Mother-baby pairs (n)	23	117	45	96	50	89
Maternal serum IgG Geometric mean titre log transformed (95% CI)	9.99 (1.16)	10.35 (0.90)	9.86 (1.51)	10.30 (1.21)	9.86 (1.79)	10.27 (1.24)
**Neonatal vaccine schedule post-dose 3**						
Maternal serum rotavirus specific IgA:						
Mother-baby pairs (n)	94	47	113	29	117	22
Maternal serum IgA Geometric mean titre log transformed (95% CI)	10.27 (1.08)	10.22 (1.20)	10.27 (1.16)	10.37 (0.94)	10.31 (1.07)	10.51 (0.80)
Maternal IgG:						
Mother-baby pairs (n)	94	46	112	29	117	22
Maternal serum IgG Geometric mean titre log transformed (95% CI)	10.25 (0.96)	10.38 (0.92)	10.08 (1.43)	10.47 (0.76)	10.04 (1.54)	10.55 (0.85)
	**SERUM IgA RESPONSE**		**SERUM IgA RESPONSE**		**SERUM IgA RESPONSE**	
	**Yes**	**No**	**Yes**	**No**	**Yes**	**No**
**Neonatal vaccine schedule post-dose 1**						
Maternal serum rotavirus specific IgA:						
Mother-baby pairs (n)	12	129	32	108	30	108
Maternal serum IgA Geometric mean titre log transformed (95% CI)	10.37 (1.26)	10.27 (1.08)	10.12 (1.25)	10.28 (1.13)	10.39 (1.13)	10.32 (1.01)
Maternal IgG:						
Mother-baby pairs (n)	12	128	32*	107	30	108
Maternal serum IgG Geometric mean titre log transformed (95% CI)	9.95 (1.07)	10.32 (0.93)	9.70 (1.64)	10.30 (1.2)	9.75 (1.62)	10.22 (1.42)
**Neonatal vaccine schedule post-dose 3**						
Maternal serum rotavirus specific IgA:						
Mother-baby pairs (n)	57	81	80	60	78*	60
Maternal serum IgA Geometric mean titre log transformed (95% CI)	10.11 (1.06)	10.41 (1.12)	10.15 (1.19)	10.36 (1.10)	10.17 (1.12)	10.56 (0.86)
Maternal IgG:						
Mother-baby pairs (n)	57*	80	79*	60	78**	60
Maternal serum IgG Geometric mean titre log transformed (95% CI)	10.06 (0.89)	10.44 (0.97)	9.93 (1.55)	10.42 (0.9)	9.81 (1.74)	10.56 (0.89)
	**SHEDDING**		**SHEDDING**		**SHEDDING**	
	**Yes**	**No**	**Yes**	**No**	**Yes**	**No**
**Neonatal vaccine schedule post-dose 1**						
Maternal serum rotavirus specific IgA:						
Mother-baby pairs (n)	12	112	15	105	29	90
Maternal serum IgA Geometric mean titre log transformed (95% CI)	10.14 (0.96)	10.33 (1.09)	10.48 (0.9)	10.17 (1.19)	10.48 (1.15)	10.27 (0.97)
Maternal IgG:						
Mother-baby pairs (n)	12	112	15	104	29	90
Maternal serum IgG Geometric mean titre log transformed (95% CI)	10.10 (1.25)	10.30 (0.90)	10.26 (1.00)	10.13 (1.44)	10.03 (1.72)	10.11 (1.50)
**Neonatal vaccine schedule post-dose 3**						
Maternal serum rotavirus specific IgA:						
Mother-baby pairs (n)	62	41	70	40	84	25
Maternal serum IgA Geometric mean titre log transformed (95% CI)	10.44 (1.06)	10.14 (1.18)	10.47 (1.21)	10.05 (1.14)	10.40 (1.07)	10.23 (1.01)
Maternal IgG:						
Mother-baby pairs (n)	62	41	70	39	84	25
Maternal serum IgG Geometric mean titre log transformed (95% CI)	10.40 (0.99)	10.24 (1.04)	10.24 (1.36)	10.25 (0.76)	10.05 (1.77)	10.28(0.77)
